# Role of *ACE2* and *TMPRSS2* polymorphisms in clinical severity and outcomes of COVID-19 in Egypt

**DOI:** 10.4102/ajlm.v13i1.2375

**Published:** 2024-08-27

**Authors:** Walaa Samy, Osama A. Gaber, Rania M. Amer, Nahawand A. El-Deeb, Ahmed A. Abdelmoaty, Ahmed L. Sharaf, Ahmed M. El-Gebaly, Rasha Mosbah, Maha E. Alsadik, Amal Fawzy, Alshymaa A. Ahmed

**Affiliations:** 1 Department of Medical Biochemistry, Faculty of Medicine, Zagazig University, Zagazig, Egypt; 2 Department of Medical Microbiology and Immunology, Faculty of Medicine, Zagazig University, Zagazig, Egypt; 3 Department of Internal Medicine, Faculty of Medicine, Zagazig University, Zagazig, Egypt; 4 Department of Tropical Medicine, Faculty of Medicine, Zagazig University, Zagazig, Egypt; 5 Department of Infection Prevention and Control, Zagazig University Hospitals, Zagazig University, Zagazig, Egypt; 6 Chest Department, Faculty of Medicine, Zagazig University, Zagazig, Egypt; 7 Department of Clinical Pathology, Faculty of Medicine, Zagazig University, Zagazig, Egypt

**Keywords:** *ACE2* gene, *TMPRSS2* gene, single nucleotide polymorphisms, COVID-19, severity, outcome

## Abstract

**Background:**

The clinical presentations of coronavirus disease 2019 (COVID-19) exhibit significant variation, ranging from asymptomatic cases to mortality resulting from severe pneumonia. Host genetics can partially explain this variation.

**Objective:**

This study evaluated possible associations between severity and outcome of COVID-19 and single nucleotide polymorphism (SNP) rs2285666 in the *ACE2* gene and SNP rs2070788 in the *TMPRSS2* gene.

**Methods:**

The study included a sample of 100 consecutive adult patients admitted to the COVID-19 Isolation and Intensive Care Units of the Zagazig University Hospitals, Zagazig, Egypt from July 2021 to November 2021. For rs2285666, polymerase chain reaction-restriction fragment length polymorphism was carried out. For rs2070788, real-time polymerase chain reaction was performed.

**Results:**

For rs2285666, the GA genotype was the most frequent among female patients (39% [16/41]) and the A genotype was more prevalent among male patients (54.2% [32/59]). For rs2070788, the AA genotype was the most frequent among all patients (46% [46/100]). No rs2285666 or rs2070788 genotypes or allele frequencies had significant associations with either severity or outcomes of patients.

**Conclusion:**

This study found no significant associations of COVID-19 severity or outcomes of patients with genotypes or allele frequencies of the rs2285666 SNP in the *ACE2* gene or the rs2070788 SNP of the *TMPRSS2* gene. The search for other genetic associations with COVID-19 infection is still required.

**What this study adds:**

The study reveals that host genetics explain the variation observed in the disease. Specific genetic variants can confer either increased susceptibility or resistance to the disease.

## Introduction

Coronavirus disease 2019 (COVID-19), which is caused by the severe acute respiratory syndrome coronavirus 2 (SARS-CoV-2), has resulted in more than 6 million fatalities and 603 million confirmed cases since its identification in 2019 until 07 September 2022.^1^ The clinical presentations of the COVID-19 infection showed significant variation, ranging from asymptomatic cases to mortality resulting from severe pneumonia. Host genetics can account for part of this variation, as some genetic variants can confer either increased susceptibility or resistance to the disease.^2^

Similar to all viruses, SARS-CoV-2 undergoes genetic mutations over time, which can potentially affect various aspects of the virus, including its ability to spread and infect, the severity of symptoms and disease, the efficacy of vaccines, diagnostic methods, and other public health interventions. Since the outbreak of the pandemic, SARS-CoV-2 has been undergoing continuous evolution. Omicron, the most divergent variant of concern, is still evolving, both genetically and antigenically. There is a possibility that it may be able to evade the existing population immunity compared with the pre-Omicron strains. Also, Omicron prefers to involve the upper rather than the lower respiratory tract; therefore, ongoing research on the SARS-CoV-2 virus is still required.^3^

The SARS-CoV-2 virus binds to host cell surface receptors by utilising spike (S) protein to mediate the fusion of the viral envelope with the cell membrane. The S protein is composed of S1 and S2 subunits. The subunit S1 contains the receptor-binding domain, that interacts with the peptidase domain of the angiotensin-converting enzyme 2 (ACE2) protein. This step is critical for the viral entry into the cell. The ACE2 protein is a well-characterised negative regulator of the renin-angiotensin system, which catalyses the conversion of angiotensin II to the heptapeptide angiotensin (1–7), which produces the opposite effect to that of angiotensin II.^4^

The ACE2 protein plays two opposing roles in COVID-19 infection. Firstly, it acts as a potent receptor for the virus to enter the cell. Secondly, it has a protective function in maintaining the balance of angiotensin II, a hormone involved in regulating blood pressure. However, when there is an excessive buildup of angiotensin II, it can lead to the release of large amounts of cytokines, causing a cytokine storm and subsequent tissue damage.^5^ The interaction between SARS-CoV-2 and ACE2 protein receptors results in a decrease in ACE2 protein expression and a reduction in its protective function on the angiotensin II pathway. Therefore, the activation of ACE2 has been proposed as a potential therapy for COVID-19. This emphasises the significance of ongoing research on *ACE2* gene polymorphisms and their functional impacts. This is important not only because the ACE2 protein receptor is involved in the development of infections, but also because it is suggested to be a potential target for therapy.^6^ The *ACE2* gene is located on chromosome Xp22; a functional single nucleotide polymorphism (SNP) in the *ACE2* gene ‘G8790A (rs2285666)’ in intron 3 could alter mRNA splicing and so affect *ACE2* gene expression.^7^ In addition, rs2285666 is suggested to alter the binding affinity of the ACE2 protein receptor for SARS-CoV-2.^8^

Receptor binding is facilitated by the human enzyme transmembrane protease serine 2 (TMPRSS2),^9^ a family of membrane-anchored proteases expressed on the airway epithelial cell surface. It acts as a viral entry cofactor by cleavage of the viral spike S protein to S1 and S2 subunits to facilitate the interaction with the ACE2 protein receptor’s extracellular domain.^10^ Therefore, genetic variations in *ACE2* and *TMPRSS2* nucleotide sequences may change the interaction of receptor-binding domain and peptidase domains, altering the host’s susceptibility to the virus and influencing the disease’s severity and outcome. The *TMPRSS2* gene is located on chromosome 21q22. Rs2070788 is a G to A transition SNP located in intron 11–12 of the *TMPRSS2* gene and was found to be associated with reduced *TMPRSS2* gene expression.^11^

Based on that, it was proposed that the aforementioned SNPs can influence the course of COVID-19 infection. This work was performed to evaluate the possible association between the *ACE2* (rs2285666) and *TMPRSS2* (rs2070788) SNPs and clinical severity and outcomes among COVID-19 patients.

## Methods

### Ethical considerations

The Institutional Review Board of the Faculty of Medicine, Zagazig University, approved this work (ZU-IRB#6775). Participants voluntarily signed a written informed consent. Procedures were carried out in agreement with the Declaration of Helsinki. Patients’ data files and sample tubes were coded, so laboratory workers were blinded to patients’ information.

### Study design and patient criteria

This prospective cohort study was performed in the Biochemistry and Clinical Pathology departments in collaboration with the Scientific and Medical Research Center, Faculty of Medicine, Zagazig University, Zagazig, Egypt, from 01 July 2021 to 30 November 2021. The study included 100 consecutive patients, ≥ 18 years old, from both genders and the same ethnicity, admitted to the COVID-19 clinic, isolation and intensive care units of the Zagazig University Hospitals, Zagazig, Egypt. Patients who refused to participate were excluded from the study.

### Diagnosis and routine investigations

Patients were selected based on the COVID-19 case definition provided by the World Health Organization.^12^ Nasopharyngeal and oropharyngeal swabs were collected and processed according to the Centres for Disease Control guidelines.^13^ The diagnosis of COVID-19 patients was made by real-time polymerase chain reaction (PCR) of the collected swabs, at the Scientific and Medical Research Centre according to the Guidelines of the Egyptian Ministry of Health and as described previously.^14^ Cycle threshold values were used as an indicator of viral load.^15^

Routine laboratory workup data including full blood counts, liver, kidney function tests, C reactive protein (CRP), lactate dehydrogenase, plasma ferritin, troponin t, and D dimer were collected from patients’ files. Additionally, all patients underwent routine chest computed tomography scans; results were collected from patients’ files after obtaining the required permissions. The Egyptian Ministry of Health and Population management roles were applied to stratify confirmed patients based on severity. Mild cases include all symptomatic patients with no radiological signs of pneumonia. Moderate cases are those with pneumonia manifestation with positive radiological signs and do not meet the criteria of severe cases. Severe cases are those with a respiratory rate > 30, oxygen saturation at room air < 92, and chest imaging showing > 50% lesions or progressive lesions within 1 day or 2 days, while critically ill patients are severe patients who still have oxygen saturation at room air < 92 or respiratory rate > 30, despite receiving oxygen therapy.^14^

Asymptomatic, mild, and moderate cases without risk were referred to home isolation with close follow-up. Conversely, moderate cases were hospitalized in COVID-19 areas, severe cases were hospitalized in intermediate care units, while critically ill patients were hospitalized in intensive care units. Therapy was carried out according to the aforementioned guidelines. Routine laboratory workup was performed in the clinical pathology department laboratories.

Based on the severity, patients were classified as mild, moderate, or severe cases, and based on the outcome patients were classified as survived and died. Demographic criteria, history of vaccination, comorbidities, occurrence of complications, viral load, and laboratory results were compared in between each two groups.

According to the guidelines of the Egyptian Ministry of Health, parenteral prophylactic anticoagulants were prescribed for hospitalised patients to guard against cardiovascular complications. In case of suspected pulmonary embolism, as most of these patients are haemodynamically unstable, and because of the risk of medical staff exposure to infection, diagnosis was based on echocardiography and D-dimer level rather than CT-angiography.^16^

### Genotyping

According to manufacturers’ guidelines, a volume of 2 mL of ethylenediaminetetraacetic acid anti-coagulated blood was obtained for DNA extraction using the QIAamp Blood Kit (Qiagen GmbH, Hilden, Germany). For the *ACE2* SNP, rs2285666, PCR and restriction fragment length polymorphism were used with the primers: forward 5′-CAT GTG GTC AAA AGG ATA TCT-3′ and reverse 5′-AAA GTA AGG TTG GCA GAC AT-3′. Polymerase chain reaction mixture included 12.5 µL of DreamTaq Green PCR Master Mix (Thermo Scientific™, Vilnius, Lithuania), 10 pmol of forward and reverse primers, 100 ng of extracted genomic DNA, and nucleases-free water up to 25 µL. The thermal cycling conditions commenced with an initial denaturation at 95 °C for 5 min, then 25 cycles (95 °C for 30 s, 60 °C for 40 s, and 72 °C for 30 s), ended by a final extension for 3 min at 72°C. The PCR products were digested using *Alu*I (Thermo Fisher Scientific Inc., Toronto, Ontario, Canada) at 37 °C for 3 h.^17^ The amplified and digested products were examined using 2% agarose gel electrophoresis for 30 min and visualized utilising an ultraviolet transilluminator. The amplicon size was 817 bp, after restriction; the G allele appears as one band at 817 bp, and the A allele appears as two bands at 589 bp and 228 bp. In women, the AG genotype appears as three bands at 817 bp, 589 bp, and 228 bp.^18^

For the *TMPRSS2* SNP, rs2070788, a real-time polymerase chain reaction assay was performed, SNP genotyping assay that contains the specific primer-probe mixture and TaqMan Universal PCR Master Mix were purchased from Thermo Fisher Scientific Inc. (Vilnius, Lithuania), amplification and detection were carried out on QuantStdioTM5 real-time polymerase chain reaction system (Applied Biosystems, Siemens Healthcare Diagnostics Inc, Singapore); all procedures were carried out according to manufacturers’ guidelines. The reaction mixture was as follows: 10 µL of (2X) TaqMan Universal PCR Master Mix, 20 units (0.5 µL) of the primer-probe assay, 1 µL of the extracted genomic DNA, and 8.5 µL nuclease-free water making the final volume of the mixture 20 µL. The real-time polymerase chain reaction cycling conditions were 60 °C for 2 min, initial denaturation at 95 °C for 10 min then 40 cycles of denaturation at 95 °C for 15 s, and annealing -extension at 60 °C for 1 min.^19^ Data analysis and results interpretation were carried out automatically by the instrument’s software based on the default cycle threshold cutoff.

### Data analysis

The SPSS^®^ statistical software version 22 (IBM Corp., Armonk, New York, United States) was used for data analysis. Numeric values are presented as mean ± standard deviation if it was normally distributed (age, haemoglobin concentration, and platelet counts), while for not-normally distributed data (total and differential leukocyte counts, prothrombin time, kidney function tests, liver enzymes, lactate dehydrogenase, ferritin, CRP, and cycle threshold value) the median (range) was used. Student’s *t*-test and one-way analysis of variance with least significant differences post hoc test were used to compare means; Mann-Whitney U test and Krusskal-Wallis with Bonferroni post hoc tests were used for medians comparison. Frequencies were expressed as numbers (percentages). Chi-square test and the two-tailed Fisher’s exact test for results ≤ 5 were used to compare frequencies. *p*-values < 0.05 were considered statistically significant differences. After comparing all variables either qualitatively or quantitatively between different groups, all variables that have *p* < 0.1 were tested by the multivariate logistic regression, and significant results are presented as *p*-value, odds ratio and 95% confidence interval. Because *ACE2* is an X-linked gene, *ACE2* G8790A genotypes were analysed separately in female and male patients.

## Results

This study included 100 patients with COVID-19 admitted to Zagazig University Hospitals; their ages ranged from 35 years to 80 years, 59% (59/100) of them were male patients, and 41% (41/100) were female patients. Apart from smoking (36%), no other substance abuse, including alcohol, has been reported; 56% of patients had history of other medical diseases including hypertension (31%), diabetes mellitus (19%), cardiovascular diseases (14%), chronic obstructive lung diseases (8%) and malignancies (6%) (Table 1). Prior to the globalisation of the vaccination programme in Egypt, only 14% of patients had been administered the Sinopharm vaccine, while 2% had received the Oxford AstraZeneca vaccine. Among the patients, fever was observed in 95% of cases, pharyngitis in 94% of cases, and chest pain in 61% of cases; 4% were diagnosed by PCR before the appearance of symptoms. Concerning severity, 51%, 34% and 15% were severe, moderate and mild cases, while 44% died by the end of the study. Nineteen patients (19%) were complicated with secondary bacterial pneumonia; 12 (12%) were infected with methicillin-resistant *Staphylococcus aureus*, 5% with *Klebsiella pneumoniae*, and 2% with *Acinetobacter* spp. Seventeen (17%) patients had cardiovascular complications in the form of lower limb ischaemia (2%), deep vein thrombosis (7%), pulmonary embolism (5%), and cerebrovascular stroke (3%). Ten (19.6%) of the severe patients group and 7 (14.2%) of the mild or moderate group had cardiovascular complications, 10 (58.8%) of whom had comorbidities in the form of hypertension (6/10; 60%), diabetes mellitus (4/10; 40%), cardiovascular diseases (3/10; 30%) and malignancy (1/10; 10%), and 7 of whom (41.2%) did not survive.

**TABLE 1:** Patient characteristics (*N* = 100), Faculty of Medicine, Zagazig University, Zagazig, Egypt, 01 July 2021 – 30 November 2021.

Characteristic	*N*	Percentage (%)	Mean ± s.d.
**Gender**			
Male	41	41	-
Female	59	59	-
**Age (years)**	-	-	57.9 ± 14.1
**Smoking**			
Yes	36	36	-
No	64	64	-
**Comorbidities**			
Yes	56	56	-
No	44	44	-
**Vaccination**			
Yes	16	16	-
No	84	84	-
**Chest pain**			
Absent	39	39	-
Present	61	61	-
**Cough**			
Absent	5	5	-
Present	95	95	-
**Body aches**			
Absent	7	7	-
Present	93	93	-
**Fever**			
Absent	5	5	-
Present	95	95	-
**Gastrointestinal tract symptoms**			
Absent	88	88	-
Present	12	12	-
**Dyspnea**			
No	24	24	-
Mild	24	24	-
Moderate	20	20	-
Severe	32	32	-
**Pharyngitis**			
Absent	6	6	-
Present	94	94	-
**Severity**			
Mild	15	15	-
Moderate	34	34	-
Severe	51	51	-
**Outcome**			
Discharge	56	56	-
Death	44	44	-
**Complications**			
Bacterial infections	19	19	-
Cardiovascular	17	17	-

s.d., standard deviation.

There were statistically significant associations between age and severity (*p* = 0.03) (Online Supplementary Table 1). Complications occurred more frequently among severe patients (*p* < 0.001). Statistically significant increases among severe cases were observed for both kidney function markers (*p* = 0.03) and CRP (*p* < 0.001). There were no significant associations between either demographic features or laboratory data and patient outcome (Online Supplementary Table 2). A statistically significant increase in mortality rate was observed in association with severity (*p* < 0.001) and the occurrence of complications (*p* = 0.02).

For the *ACE2* SNP rs2285666, the GA genotype was the most frequent among female patients (39%), while among male patients the A genotype was more prevalent (54.2%). For the *TMPRSS2* SNP rs2070788, the AA genotype was the most frequent among all patients (46%). *ACE2* rs2285666 and *TMPRSS2* rs2070788 genotypes and allele frequencies had no significant associations with either disease severity or patient outcomes, as shown in Table 2 and Table 3. Multivariate logistic regression revealed that CRP level was the only independent predictor of COVID-19 severity in this cohort: *p* = 0.001, odds ratio (95% confidence interval) were 1.1 (1.0–1.2) (data not presented). Furthermore, it was found that severity was an independent predictor of outcome: *p* < 0.001, odds ratio (95% confidence interval) were 46.5 (10.2–211.2).

**TABLE 2:** Associations between COVID-19 severity and single nucleotide polymorphisms of the *ACE2* and *TMPRSS2* genes, Faculty of Medicine, Zagazig University, Zagazig, Egypt, 01 July 2021 – 30 November 2021.

Patients	Genotype or allele	Mild or moderate (*n* = 49) (%)		Severe (*n* = 51) (%)		OR	95% CI	*p* *
		*N*	%	*n*	%			
***ACE2* (rs228566)**								
Women (*n* = 41)		-	-	-	-	-	-	-
	GG	8	16.3	5	9.8	0.7	0.2–2.7	0.8
	GA	9	18.3	7	13.7	0.9	0.2–3.5	-
	AA	6	12.3	6	11.7	1.4	0.4–5.4	-
Men (*n* = 59)		-	-	-	-	-	-	-
	G	12	24.5	15	29.4	0.9	0.3–2.7	0.8
	A	14	28.6	18	35.3	-	-	-
All participants (*N* = 100)		-	-	-	-	-	-	-
	G	37	37.8	32	31.4	0.8	0.4–1.6	0.7
	A	35	35.7	37	36.2	-	-	-
***TMPRSS2* (rs2070788)**								
All participants (*N* = 100)		-	-	-	-	-	-	-
	GG	14	28.6	16	31.4	1.9	0.8–4.3	0.8
	GA	11	22.4	13	25.5	1.2	0.5–2.9	-
	AA	24	49.0	22	43.1	0.5	0.2–1.0	-
All participants (*N* = 100)		-	-	-	-	-	-	-
	G	39	39.7	45	44.2	0.8	0.5–1.5	0.5
	A	59	60.3	57	55.8	-	-	-

OR, odds ratio; CI, confidence interval.

* , Chi-square test.

**TABLE 3:** Associations between COVID-19 outcomes and single nucleotide polymorphisms of the *ACE2* and *TMPRSS2* genes, Faculty of Medicine, Zagazig University, Zagazig, Egypt, 01 July 2021 – 30 November 2021.

Patients	Genotype or allele	Discharge (*n* = 56)		Death (*n* = 44)		OR	95% CI	*p* *
		*n*	%	*n*	%			
***ACE2* (rs228566)**								
Women (*n* = 41)		-	-	-	-	-	-	0.2
	GG	9	16.1	4	9.1	0.8	0.2–3.3	-
	GA	8	14.3	8	18.2	3.1	0.8–12.1	-
	AA	10	17.9	2	4.5	0.2	0.1–1.5	-
Men (*n* = 59)		-	-	-	-	1.4	0.5–3.9	0.6
	G	12	21.4	15	34.1	-	-	-
	A	17	30.3	15	34.1	-	-	-
All participants (*N* = 100)		-	-	-	-	1.4	0.7–2.7	0.4
	G	38	33.9	31	35.2	-	-	-
	A	45	40.1	27	30.7	-	-	-
***TMPRSS2* (rs2070788)**								
All participants (*N* = 100)	-	-	-	-	-	-	-	0.4
	GG	17	30.3	13	29.5	0.9	0.4–2.3	-
	GA	16	28.6	8	18.2	0.5	0.2–1.4	-
	AA	23	41.1	23	52.3	1.6	0.7–3.5	-
All participants (*N* = 100)		-	-	-	-	0.8	0.4–1.4	0.3
	G	50	44.6	34	38.6	-	-	-
	A	62	55.4	54	61.4	-	-	-

OR, odds ratio; CI, confidence interval.

* , Chi-square test.

## Discussion

The mean age of patients who developed severe disease was significantly higher than mild or moderate cases. This aligns with many previous findings that age is a major risk factor for COVID-19, and the most unfavourable outcomes are attributed to age-related comorbidities and immunocompromising status.^20^ Patients with severe disease showed a slight but significant increase in serum urea and creatinine. Hachim et al.^21^ studied the kidney function in 250 COVID-19 patients in the Middle East region. The study reported an association between COVID-19 severity and renal impairment. Additionally, these results agree with a study conducted by Gabarre et al. in 2020 in Paris, France,^22^ in which 42.9% of critically ill patients developed acute kidney injury. This finding highlights the value of evaluating kidney function tests in patient monitoring.

Although it was elevated in 94% of patients, CRP showed a sharp increase in severe cases, and it was the only independent predictor for patients’ severity in this work. These results go with those of Wang et al. in 2020 in China,^23^ who reported that all patients who developed severe diseases displayed elevated CRP results and that CRP was the only independent predictor of severity. Potempa et al.^24^ revealed that CRP was a reproducible, rapid, and cost-effective marker to evaluate the extent of tissue damage and inflammatory process in COVID-19 patients.

The viral load did not significantly influence disease severity. Prior research has demonstrated conflicting findings on this topic, Cho et al. in 2020 in Hong Kong, China,^25^ reported that viral load is not associated with either severity or outcome. Abdulrahman et al.^26^ explain that the correlation between disease severity and CRP level, rather than viral load, can be attributed to the influence of host factors on disease progression and the contribution of pro-inflammatory cytokines to the severity of the disease.

The mortality rate was higher in severe cases than in mild or moderate cases, particularly in those who encountered complications during the disease course. This result agrees with previous studies in Egypt and worldwide. Gaber et al., in 2022 in Egypt,^27^ reported that oxygen saturation and chest computed tomography severity score are predictors of mortality. Furthermore, Assal et al., in 2022 in Egypt,^28^ found that the need for mechanical ventilation and the occurrence of secondary bacterial infections are associated with higher mortality. Additionally, Mahendra et al., in 2021 in India,^29^ indicated that severe pneumonia is associated with a higher fatality rate.

Angiotensin-converting enzyme 2 and *TMPRSS2* gene polymorphisms have been implicated previously in altering the susceptibility to COVID-19 infection and influencing severity and mortality rates.^30^ Wang et al., in 2020 in China,^31^ demonstrated that *ACE2* SNPs-rs4646116, rs267606406, and rs143936283 were associated with higher affinity to the receptor-binding domain. In contrast, *ACE2* SNPs-rs1244687367, rs146676783, and rs961360700 were associated with lower binding affinity and hence decreased susceptibility to COVID-19 infection.

Also, *TMPRSS2* gene polymorphisms have been associated with susceptibility to COVID-19 infection and outcome.^32^ In a study on Italian patients in 2020, Asselta et al. revealed that *TMPRSS2* SNPs-rs2070788, rs9974589, and rs7364083 were associated with increased severity of COVID-19.^32^ Moreover, Fuentes et al. reported that rs61735794 and rs61735792 were significantly associated with COVID-19 infection outcome.^33^ In this work, no significant associations were detected between either the *ACE2* rs2285666 SNP or the *TMPRSS2* rs2070788 SNP and COVID-19 severity and outcome.

The AA genotype of rs2285666 (G8790A) was found to increase *ACE2* gene expression up to 50%.^34^ However, controversies have been observed regarding the effect of this point mutation; Srivastava et al., in 2020 in India,^35^ demonstrated a negative correlation between the alternate allele (A) and case fatality rate of SARS-CoV-2 infection, and Möhlendick et al., in 2021 in Germany,^36^ reported a threefold increase in severity in cases carrying the GG genotype. Hence, they suggested that rs2285666 harbours a protective role against COVID-19. A possible explanation for this finding is that the increased serum and tissue-bound ACE2 protein in patients carrying the AA genotype counteracts the pro-inflammatory and fibrotic effects of angiotensin II.^37^

However, Martínez-Gómez et al., in 2022 in Mexico,^38^ and Sabater et al., in 2022 in Spain,^39^ found that the AA genotype was associated with critical outcome. The current work’s results agree with those of Alimoradi et al., in 2022 in Iran,^18^ Karakaş et al., in 2021 in Turkey,^40^ and Elnagdy et al., in 2022 in Egypt,^41^ that there is no significant association between rs2285666 and COVID-19 severity and outcome. Variations in findings between studies can be attributed to factors such as ethnicity variations, sample size, patients’ habits, and, notably, viral load. In addition, along with genetic variations, many other factors can influence the balance between the susceptibility and protective roles of ACE2 protein, such as smoking, diet, comorbidities, age, sex, and cleavage of the ACE2 protein by products of the *TMPRSS2* and *ADAM17* genes.^5^

Pandey et al., in 2022 in India,^42^ and Asselta et al., in 2020 in Italy,^32^ observed that the *TMPRSS2* SNP rs2070788 had a statistically significant association with disease severity and fatality rate in American and Italian populations, respectively. Another multicentre study by Irham et al. in 2020^11^ showed that rs2070788 and another three SNPs, which affect the expression of the *TMPRSS2* gene in lung tissues, were more prevalent in American and European populations than in Asian populations; this finding can explain the higher susceptibility and fatality rates in American and European populations. Schönfelder et al., in 2021 in Germany,^43^ observed no significant association between rs2070788 and COVID-19 severity. In addition, they attributed differences among several studies to variations in sample size, ethnicity, and the possibility of the presence of linkage disequilibrium with rs383510.

### Limitations

This study has some limitations that should be considered when interpreting the findings. Firstly, this was a single-centre study with a relatively low sample size. Thus, the results may be insufficiently powered and require further confirmation by future multicentre studies with larger cohorts. Secondly, host genetics is not the only factor that can influence COVID-19 disease: the host immunological status as well as SARS-CoV-2 strain variations have impacts on disease severity and outcome. In addition, an interplay between all of these factors exists; no data were available about patients’ antibody response or viral genotypes so these factors could not be included in the analyses. This may have resulted in insufficient data about the impact of these factors on disease behaviour.

### Conclusion

The genotypes and allele frequencies of the *ACE2* gene SNP rs2285666 and the *TMPRSS2* gene SNP rs2070788 showed no significant associations with the severity and outcome of patients. Further investigation into additional genetic correlations with COVID-19 infection is still necessary.
